# A Study of a Handrim-Activated Power-Assist Wheelchair Based on a Non-Contact Torque Sensor

**DOI:** 10.3390/s16081251

**Published:** 2016-08-08

**Authors:** Ki-Tae Nam, Dae-Jin Jang, Yong Chol Kim, Yoon Heo, Eung-Pyo Hong

**Affiliations:** Korea Orthopedics & Rehabilitation Engineering Center, 26, Gyeongin-ro 10beon-gil, Bupyeong-gu, Incheon 21417, Korea; ktnam@kcomwel.or.kr (K.-T.N.); djjang@kcomwel.or.kr (D.-J.J.); yckim@kcomwel.or.kr (Y.C.K.); heruni1004@kcomwel.or.kr (Y.H.)

**Keywords:** non-contact torque sensor, assistive technologies, power wheelchair, handrim-activated

## Abstract

Demand for wheelchairs is increasing with growing numbers of aged and disabled persons. Manual wheelchairs are the most commonly used assistive device for mobility because they are convenient to transport. Manual wheelchairs have several advantages but are not easy to use for the elderly or those who lack muscular strength. Therefore, handrim-activated power-assist wheelchairs (HAPAW) that can aid driving power with a motor by detecting user driving intentions through the handrim are being researched. This research will be on HAPAW that judge user driving intentions by using non-contact torque sensors. To deliver the desired motion, which is sensed from handrim rotation relative to a fixed controller, a new driving wheel mechanism is designed by applying a non-contact torque sensor, and corresponding torques are simulated. Torques are measured by a driving wheel prototype and compared with simulation results. The HAPAW prototype was developed using the wheels and a driving control algorithm that uses left and right input torques and time differences are used to check if the non-contact torque sensor can distinguish users’ driving intentions. Through this procedure, it was confirmed that the proposed sensor can be used effectively in HAPAW.

## 1. Introduction

Research and development of new products are continuously being performed on handrim-activated power-assist wheelchairs (HAPAW), which were first commercialized in the 1990s [1,2,3,4,5]. Research on a power-assist wheelchair (PAW), which detects the caregiver’s driving intention through a handle instead of the handrim to assist mobility, has also taken place [5]. The demand for high-performance mobile devices such as PAW is increasing with global aging and enhancement of living standards. Therefore, research on assistive technology such as PAW is becoming more important.

HAPAW has the advantages of manual wheelchairs, such as lightness and convenient storage and transportation, along with the advantages of electric-powered wheelchairs such as drivability on steep paths or over long distances. The elderly and disabled have difficulty in moving freely by themselves over bumps or tilted surfaces with conventional manual wheelchairs [6,7]. To address this challenge, HAPAW must be designed to detect the propulsion of the user that is applied to the handrim and assist the driving torque of the wheelchair to help users who lack strength to drive over long distances or on steep paths. It has been reported that HAPAW is effective for a person who has difficulty in handling a wheelchair or who does not have sufficient strength to handle it [8].

HAPAW measures users’ driving intentions applied to the left and right rotating handrims, and it is important to effectively deliver the measured signals to the controller located in the body frame that does not rotate. JWII (Yamaha Motor Corp., Shizuoka, Japan) uses a special circular connector to transfer signals from the rotating handrims to the fixed controller [3]. e-Motion (Alber GmbH, Albstadt, Germany) solved wire distortion by rotating the battery and controller, which were embedded in the wheel along with the handrim [2]. Sensorless HAPAW, which does not use torque sensors, is also being researched [9,10], but more research is required for commercialization owing to vulnerability to disturbances. Recent studies aimed at accurately measuring the driving characteristics of a wheelchair or analyze its torque and kinetic energy [11,12] are remarkable in HAPAW research.

The torque applied to the left and right handrims of HAPAW is uneven owing to physical features or disability, which makes the driving wheel output of HAPAW unstable; this causes degradation of wheelchair driving performance. Control methods to properly match user characteristics and road conditions are required because straight and rotating driving are the most basic driving performances. The mutual reference method, which refers left and right assist torques to the opposite propulsion torque, is used to solve this problem [13,14,15]. It is important in the mutual reference method to maintain rotational performance along with the improvement of straight performance.

A driving wheel that uses a non-contact torque sensor was designed and prototyped in this study to achieve users’ driving intentions. A driving wheel mechanism with an embedded non-contact torque sensor was designed, and the torque value applied to the handrim by external forces was simulated. After manufacturing a driving wheel prototype, torque measurement tests were conducted and it was confirmed that torques are similar to simulation values. A balancing algorithm that enhances straight and rotation driving performance by using input torques and their time differences [13] was used to check if the prototyped driving wheel could be applied in HAPAW by testing whether the input torque was balanced. Finally, the applicability of the designed driving wheel in HAPAW was confirmed.

## 2. Materials and Methods

### 2.1. Design of a Driving Wheel with a Non-Contact Torque Sensor

**Figure 1:**
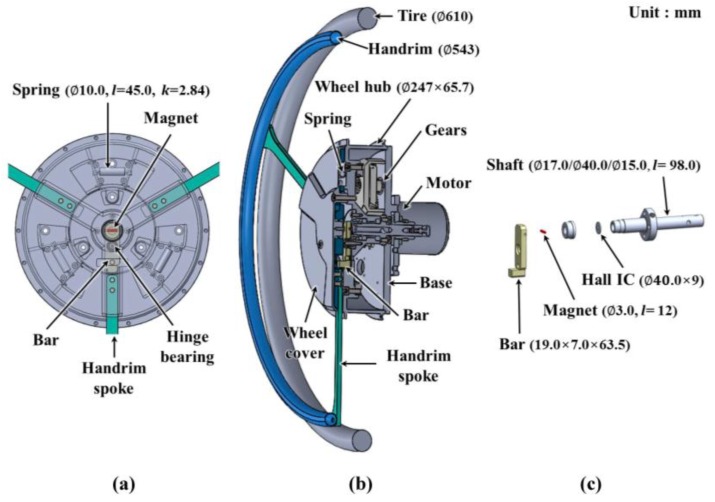
Designed wheel using a magnetic non-contact torque sensor. (**a**) The handrim system moving back and forth by 8° increments flexibly; (**b**) the driving wheel including the non-contact torque sensor and handrim system; and (**c**) the parts of the non-contact torque sensor, including a magnet and a Hall IC. The bar including the magnet moves with the handrim, and the Hall IC outputs a signal that is directly proportional to the magnetic field.

A sensor that measures the force applied to the handrim is essential in HAPAW. The wires for sensor power and signal transmission become entangled because the handrim rotates with the wheel. This increases the price when special devices are used or when the structure needed to solve this problem becomes too complex. The non-contact torque sensor applied in the HAPAW driving wheel shown in Figure 1 was designed in this study to measure user input torque while maintaining a simple structure. As shown in Figure 1, the driving wheel is composed of a motor, gears, wheel hub, handrim restoration device, and sensor. Available commercial HAPAW have handrims that allow flexible movement in some intervals back and forth as the handrim moves. The handrim system in this study was designed to allow flexible movement by the spring in 8° increments, and the control system can detect the user’s driving intention and assist the driving force required within this regime.

As shown in Figure 1a, the designed handrim system arranges three handrim spokes with a coiled tension spring at 120° intervals to implement flexible movement. The components of the handrim system are protected by a wheel cover and connected with the wheel hub. As shown in Figure 1, the motor, gears, and shaft are fixed to the base and connected to the body frame, which does not rotate. The wheel hub and the handrim fixed to the wheel hub rotate with the tire. As shown in Figure 1c, the proposed sensor is composed of a bar that has a hinge structure that moves in proportion to handrim movement, a magnet fixed to the bar, and a Hall IC fixed at the center of the shaft to detect the movement of this magnet. The handrim spoke moves when the handrim moves, and the bar that is separated from the handrim spoke also moves. The magnet moves as the bar moves, and the Hall IC senses this movement to detect the driving intentions of users.

**Figure 2:**
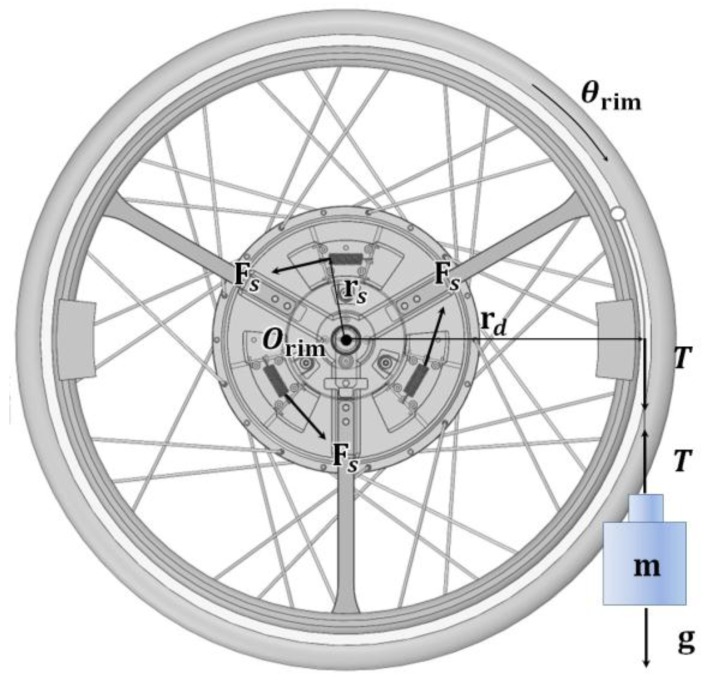
Free body diagram for driving torque measurement of the new designed wheel.

To investigate the user input torque in the assisting wheel, the torque values measured by the designed wheel mechanism were simulated. Figure 2 shows the free body diagram of the designed wheel. The user’s driving torque can be calculated with the rotational moment of inertia and the moment of the internal spring force when the user moves the handrim. When applying an external force T to the handrim, which has radius $r_{d}$, the driving torque τ is:

(1) $$\tau =r_{d}T$$

and is equivalent to the sum of the rotational moment of inertia and the moment of the internal spring force, which is expressed as follows:

(2) $$\tau =I\alpha +3r_{s}F_{s}$$

Here, $r_{s}$ is the distance from the center of rotation of the handrim to the spring center line, $F_{s}$ is the spring force that occurs during handrim rotation, I is the inertia moment, and α  is the angular acceleration of the handrim as shown in Figure 2.

Inertia moment I is calculated using the axis that passes the rotation center point of the handrim, $O_{\text{rim}}$. The result calculated with the design program SolidWorks (Dassault Systèmes, Waltham, MA, USA) was 3.92 × 10^−2^ kg·m^2^. $F_{s}$ is calculated by the handrim angle as shown in Equation (3):

(3) $$F_{s}=kdx+F_{pre}=kr_{s}\theta_{\text{rim}}+F_{pre}$$

Here, k is the spring constant, dx is the length variation of the spring,$\;\theta_{\text{rim}}$ is the angle of handrim rotation, and $F_{pre}\;$ is the initial tension of the extension spring. $\theta_{\text{rim}}$ in Equation (3) is reasonable because the rotation range is small. Equation (4) is derived when substituting Equation (3) into Equation (2):

(4) $$\tau =I{\ddot{\theta }}_{\text{rim}}+3kr_{S}^{2}\theta_{\text{rim}}+3r_{s}F_{pre}$$

**Figure 3:**
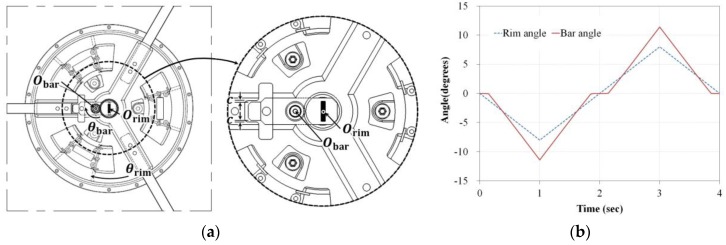
(**a**) Shape of movement of the bar and (**b**) simulation results of the rim and bar angles.

The handrim is rotated about the point $O_{\text{rim}}$ as shown in Figure 3, but the sensor bar is rotated about the point $O_{\text{bar}},$ which is slightly more distant from the center of rotation of the handrim. Therefore, an equation relating the rotation angle of the handrim $\theta_{\text{rim}}$ and the rotation angle of the bar $\theta_{\text{bar}}$ is required, and the following equation was obtained through numerical simulation:

(5) $$\theta_{\text{rim}}=0.598\theta_{\text{bar}}+1.16$$

The bar started to move when the handrim moved above 1°, as shown in Figure 3a because of the clearance c between the wheel hub and bar. The maximum handrim angle is 8° and the maximum bar value is approximately 11.4°, as shown in Figure 3b.

A push–pull gauge (DS2-200N, IMADA, Aichi, Japan) was used to calculate the spring constant by rotating the handrim in 1° increments from zero to 8°, and the external force was measured while the handrim was maintained in a certain position. Angular acceleration becomes 0 when the handrim stops, as Equation (4) shows above.

(6) $$\tau =3kr_{S}^{2}\theta_{\text{rim}}+3r_{s}F_{pre}$$

**Figure 4:**
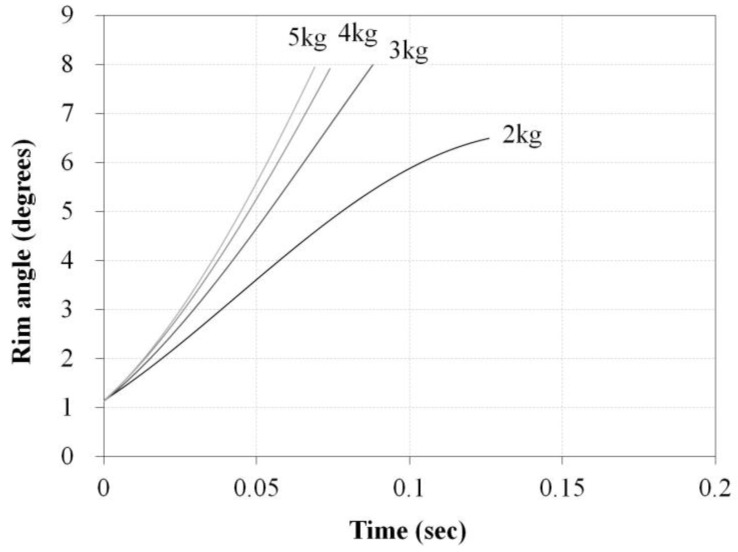
Simulation results for bar angle with extension springs for 2~5 kg dropping weights.

**Figure 5:**
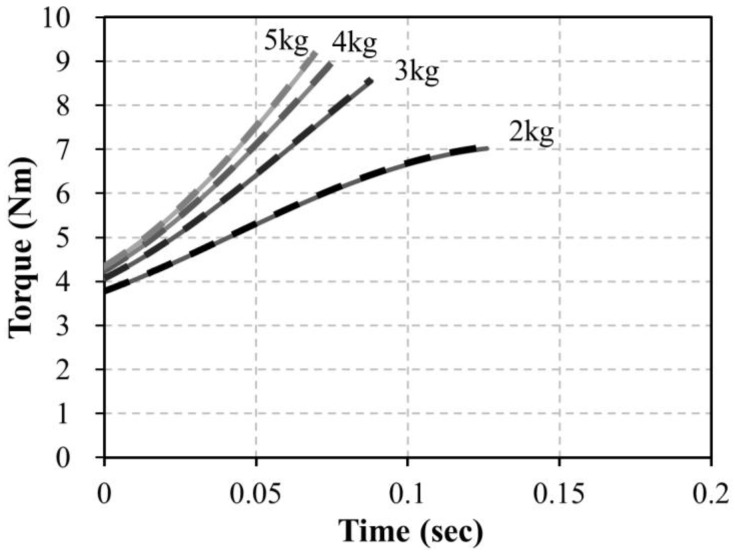
Simulation results for the driving torque in Equation (1) (dashed line) and calculated torque in Equation (2) (solid line) with extension springs for 2~5 kg dropping weights.

As a result of the test, the spring constant k was found to be 2.84 N/mm and the initial spring tension $F_{pre}$ was 5.45 N. These values were used to simulate the driving torque. As shown in Figure 2, the angular variation of the bar when dropping 2~5 kg weights was simulated through the Adams 12.0 (MSC Software, Newport Beach, CA, USA) program, and the results shown in Figure 4 were obtained. The driving torque shown in Equation (1) generated by the external force and the calculated torque in Equation (2) corresponded with the simulation results shown in Figure 5. Through these results, it was confirmed that the torque value calculated by the handrim angle $\theta_{\text{rim}}$ can be used when driving the handrim.

### 2.2. Generation of the Handrim-Activated Wheelchair Movement

Various studies are being performed for HAPAW control [16,17], and the most basic control functions for a wheelchair are driving straight and rotation. A stabilized input torque must be applied to the left and right side handrims to maintain a constant driving direction. However, the strengths of both arms of wheelchair users are not generally equal, and it is not easy to generate well-balanced left and right torques. Therefore, a control method that can stably generate balanced torques from the uneven input torques of users is needed to maintain a stable driving direction for HAPAW [13,14,15].

**Figure 6:**
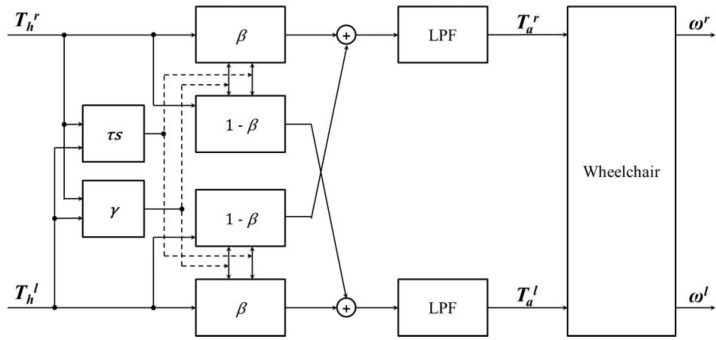
Configuration of system structure for balanced torque generation, where the direction of balanced torques is the same as those of the input torques [13].

To check if the torque sensor and driving wheel manufactured in this study are proper for applications in HAPAW, left and right input torques and time difference were used for the control method that adjusts the balance ratio so that it is suitable for driving straight and rotation [13]. Figure 6 shows the diagram of the control system used. It generates right and left balanced torques $T_{b}^{r}$ and $T_{b}^{l}$ from the right and left human torques $T_{h}^{r}$ and $T_{h}^{l}$. It is known that the assist torques Tar and Tal passed through a low-pass-filter (LPF) are applied to the mechanical wheelchair. In Figure 6, *β* is the balance ratio, *τs* is the temporal similarity between the left and right input torques, and the range is expressed in values between 0 and 1. *γ* is the ratio of the two torques, and has a value between −1.0 and 1.0. The balanced torques *Tbr* and *Tbl* in Figure 6 are calculated as follows:

(7) $$T_{b}^{r}=\;dir^{r}\left(\beta \left|T_{h}^{r}\right|+\left(1-\beta \right)\left|T_{h}^{r}\right|\right)$$

$$T_{b}^{l}=dir^{l}\left(\beta \left|T_{h}^{l}\right|+\left(1-\beta \right)\left|T_{h}^{l}\right|\right)$$

These satisfy 0.5 ≤ *β* ≤ 1 and [13] $dir^{r}$ and $dir^{l}$ are the directions of the balanced torques equivalent to those of the input torques. For example, the output torque reflects 50% of the opposite input torque when *β* in Equation (7) reaches 0.5, making it easy to drive straight. On the other hand, if *β* approaches 1, the opposite input torque is not reflected, making rotation easy.

**Figure 7:**
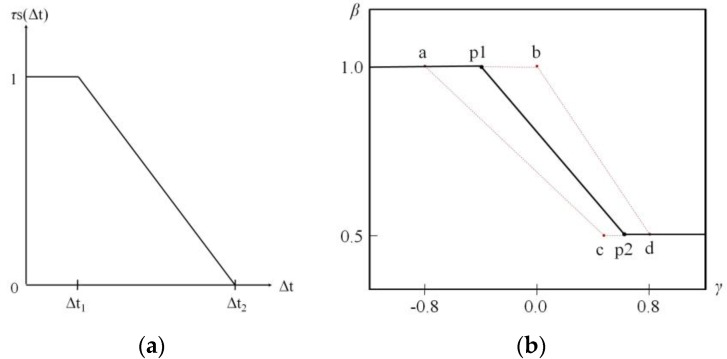
(**a**) The temporal similarity function and (**b**) the balance ratio design with its variation range [13]. a, b, c, and d are coordinates for the *γ* and *β* axes used in this study; these are (−0.8, 1.0), (0, 1.0), (0.5, 0.5), and (0.8, 0.5), respectively.

Figure 7 shows the temporal similarity function (TSF) and the range of *β* that uses this function. Figure 7a is the TSF that judges the temporal similarity between the left and right propulsion torques. Figure 7b shows *β*, which fluctuates according to TSF results, and the predefined inflection points a, b, c, and d show the fluctuation range of *β*. A time difference between left and right input torques smaller than Δt1 in Figure 7a means that the temporal similarity is very large, indicating that the user’s drive intention is to drive straight. In this case, inflection points p1 and p2 are set to be close to points a and c, respectively, regarding the setting range of *β*, and a modification is conducted to enhance straight-driving performance even if input torques with large left and right differences occur. In this study, when inflection points p1 and p2 approach a and c, respectively, *β* is set to be 0.5 even if the left and right torque proportion falls to 50%.

On the other hand, the driving intention of the user can be judged to be closer to rotation than driving straight if the time difference between the left and right propulsion torques is larger than Δt2. At this time, the controller brings inflection points p1 and p2 close to points b and d, respectively, to enhance direction change performance as shown in Figure 7b. Thus, the controller generates independent output torques from the opposite side input torques.

The sensitivity of the balance controller for detecting the driving intentions of a user is determined by the values of Δt1 and Δt2. With increasing Δt1 and Δt2, straight sensitivity is increased as increasing time for referencing the left and right torques. With decreasing Δt1 and Δt2, rotation sensitivity is increased because of the torque applied independently to the wheel. The average driving speed of manual wheelchair users in daily life is approximately 0.79 m/s [18], and it is reported that the frequency of pushing handrims is less than 1 Hz and that wheelchair speed does not exceed 0.9 m/s [19]. The time difference between left and right propulsion torque signals is within 100 ms if the driving intention of a user is to drive straight [13]. Following this condition, Δt1 and Δt2 for the TSF each must be set to be larger than 100 ms and smaller than 1 s. In this study, Δt1 was set as 200 ms and Δt2 was set as 400 ms.

**Figure 8:**
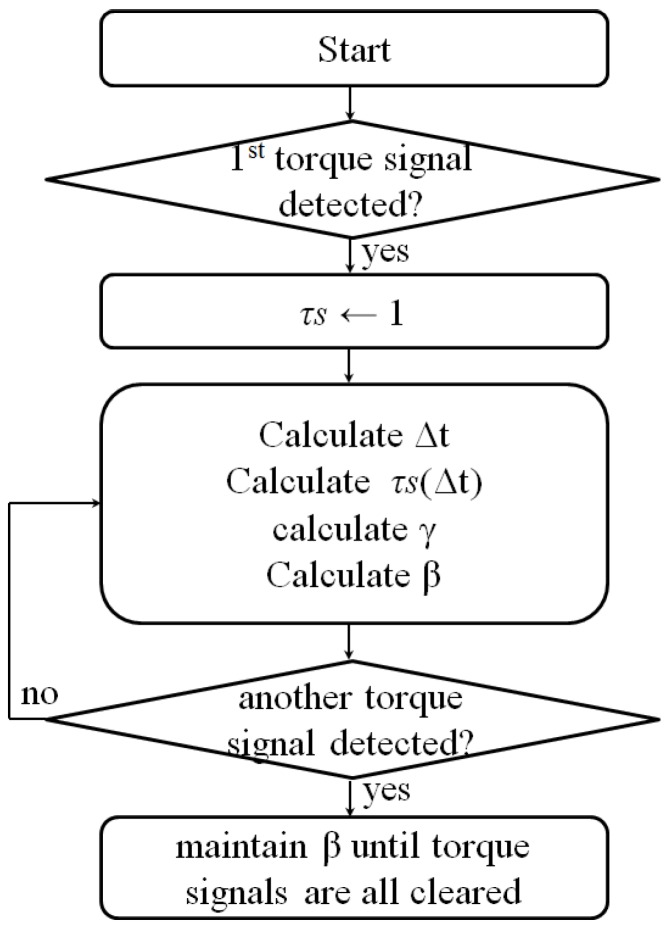
Decision process for temporal similarity and balance ratio.

Figure 8 shows the process of determining the temporal similarity and *β*. As shown in Figure 7a, when a signal is detected from the input torque sensor, *τs* is first set to have a maximum value of 1.0 and decreases as time passes until the input signal on the opposite side is detected. When the input torque on the opposite side is detected, the *τs* value at this time indicates the temporal similarity, and that value is maintained until input torques on both sides disappear. It is used as a coefficient that controls *β* for generation of the assist torque.

**Figure 9:**
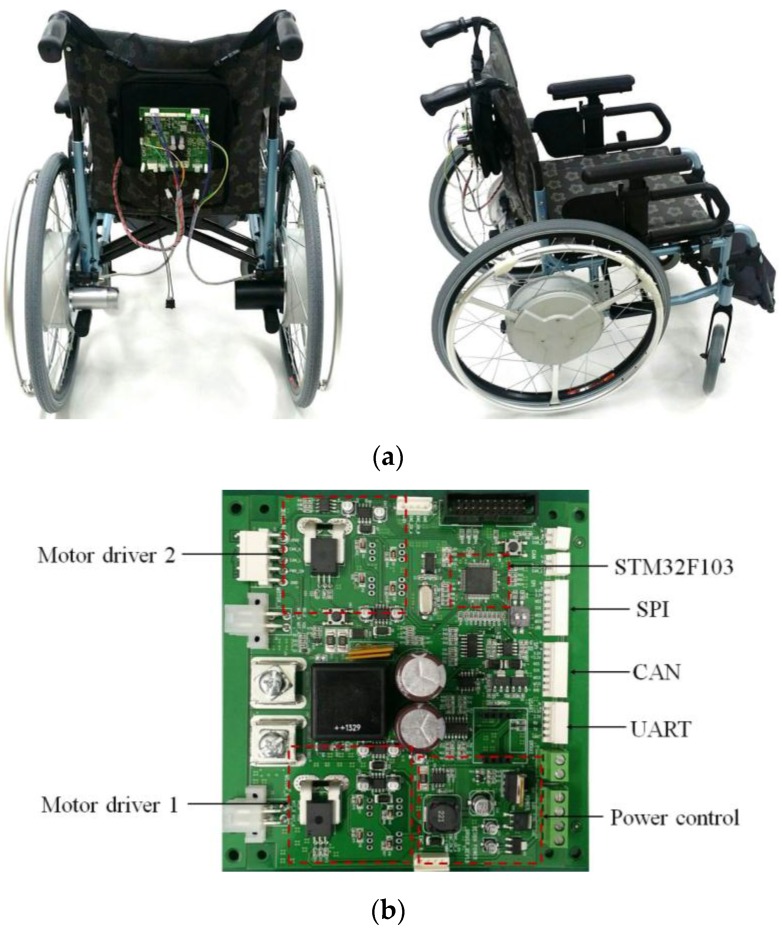
Prototype of the proposed device. (**a**) The assembled HAPAW and (**b**) the implemented motor driver.

A HAPAW prototype was developed for driving tests to verify the usefulness of the proposed torque sensor, and the related specifications are shown in Table 1. The magnet used in the torque sensor was a model 400300401 (Standex-Meder Electronics, Cincinnati, OH, USA), and the tension spring was a AWF10-45 (Misumi Group Inc., Tokyo, Japan). The Hall IC used to measure magnetic flux was a model WSH136 (Winson Semiconductor Corp., Hsin-Chu, Taiwan). Under the supplied power of +5 V, WSH136 shows linear characteristics within the range −700 G to +700 G with a sensitivity of 3.0 mV/G. The HAPAW system is shown in Figure 9 and is composed of the driving wheels, controller, and battery. The battery used in the prototype was a 24 V, 8 Ah lithium ion battery. Although the battery capacity, the weight of driving wheel, and the total weight of the wheelchair are the main specifications of HAPAW, we did not consider them significant when developing the prototype. The driving wheel control MCU was a Corex-M3 type STM32F103RF (STMicroelectronics, Fairport, NY, USA) and an IRF1402 (Infineon, Munich, Germany) for power switching was applied. Bluetooth made it possible to obtain the control status or transfer commands through wireless communication.

## 3. Results

**Figure 10:**
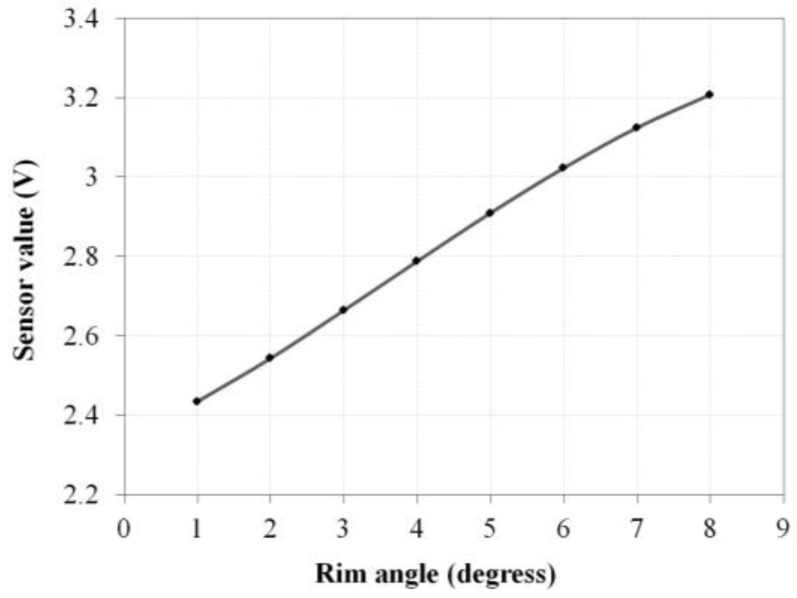
Results of average Hall sensor voltage measured at 1° increments.

**Figure 11:**
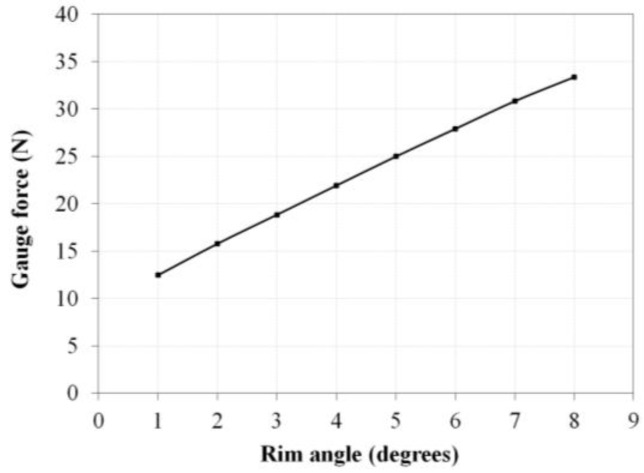
Results of average pull force measured by the push–pull gauge at 1° increments.

Figure 10 shows the results of measured Hall sensor output values according to handrim angle. Linearity is observed in the 2–6° handrim angle range, but some non-linearity is observed at the ends. External force was measured 10 times as the handrim angle was changed by 1° increments between 1° and 8°, and the mean value of the spring constant was calculated. The push–pull gauge measurement results are shown in Figure 11 and the spring constant k and initial spring tension $F_{pre}$ are calculated from Equations (1) and (6). The results showed the spring constant k to be 2.84 N/mm and the initial spring tension $F_{pre}$ to be 5.45 N.

**Figure 12:**
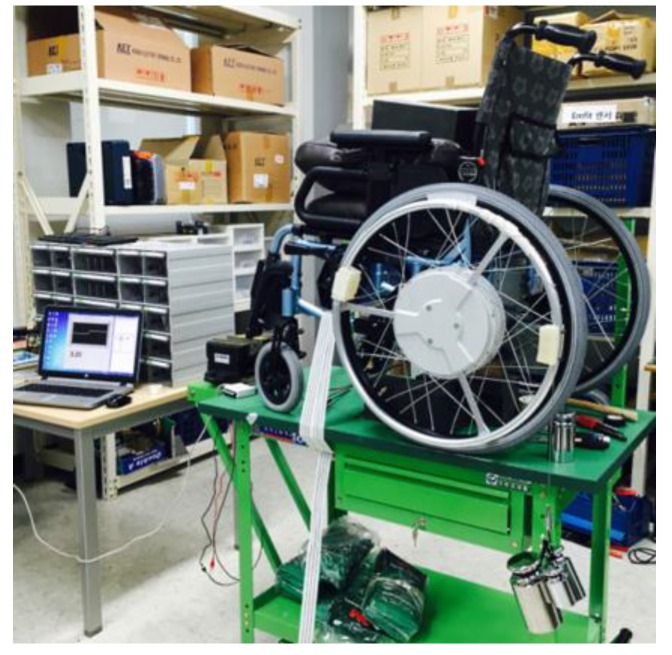
Picture of the experiment dropping various weights.

The experiment of dropping actual weights of 2–5 kg was conducted with the manufactured driving wheel to compare with the simulated torques. Three trials of dropping each weight were performed, and the angle of the handrim was measured by the voltage of the Hall IC. The sampling rate of the Hall IC was 1 kHz and the measured Hall IC voltage values were converted into handrim angle values. Figure 12 shows a picture of a dropping weight. After placing a 60 kg dummy on the HAPAW sheet and fixing the wheels to prevent movement during the test, the dropping weight test was conducted with varying weights.

**Figure 13:**
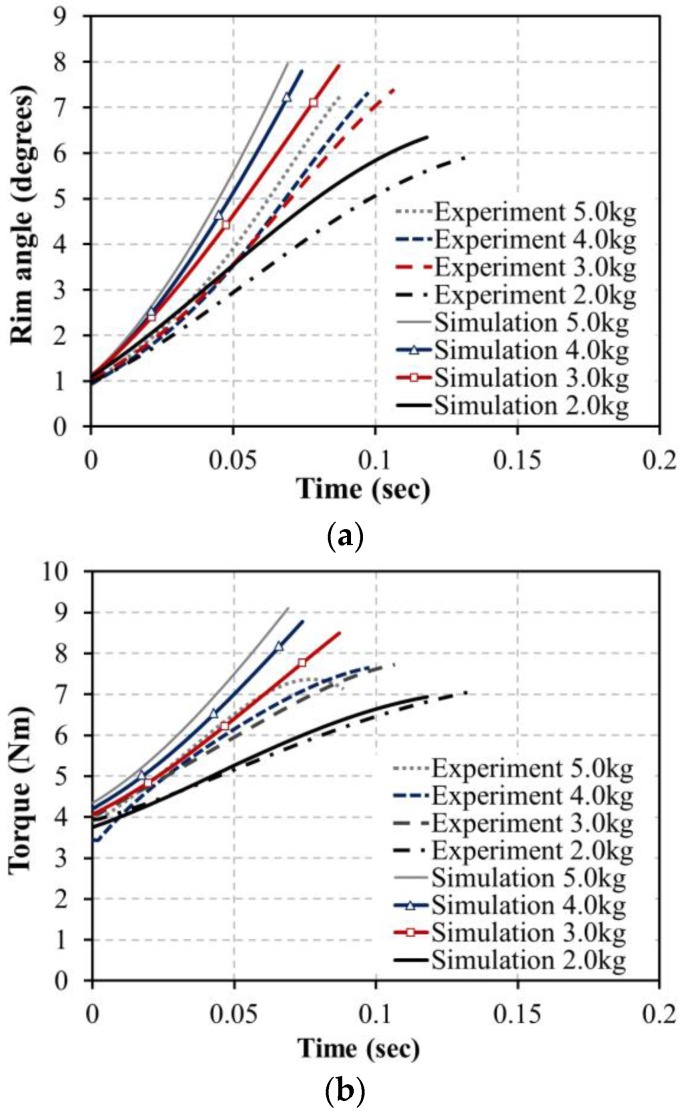
Comparison of simulations and experiments in the dropping weight test for (**a**) handrim angle and (**b**) torque.

Figure 13 shows the results of the experiment of dropping various weights. As a result of the experiment, it is clear from the handrim angle in Figure 13a that simulation angles increase more quickly than actual test values. This is considered to be due to the air resistance during falling, friction between the handrim and wheel hub, and non-ideal spring movement, which showed slower increases than the simulation values. However, the increasing angle of the handrim with increasing weight was shown to have a similar tendency in the simulation and test results. Along with the handrim angle results, the driving torque value in Figure 13b was shown to be approximately 10% lower in the actual test compared to the simulations, and this is judged to be due to similar reasons as for the handrim angle results.

**Figure 14:**
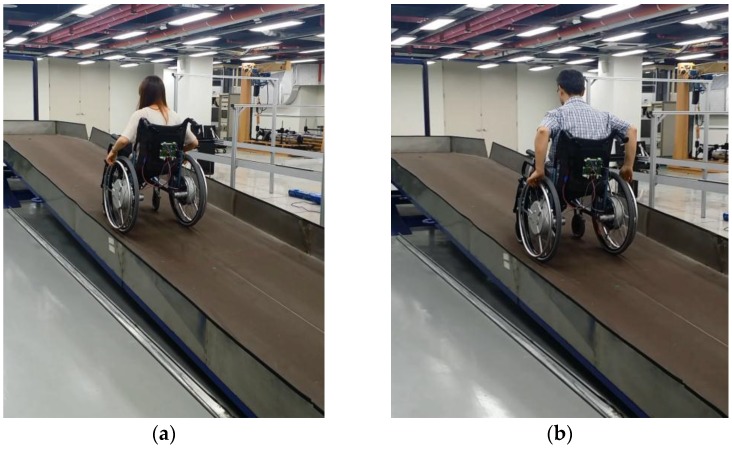
Driving on a ramp with (**a**) a female user and (**b**) a 100 kg user.

As shown in Figure 14, driving tests were performed with the HAPAW on a ramp to test the operation characteristics of the developed non-contact torque sensor. A female user without wheelchair experience could easily operate the wheelchair on a ramp. A 100 kg user could easily drive up the ramp sufficiently. Therefore, it was confirmed that the measured driving torque from the driving wheel could be used in actual applications.

**Figure 15:**
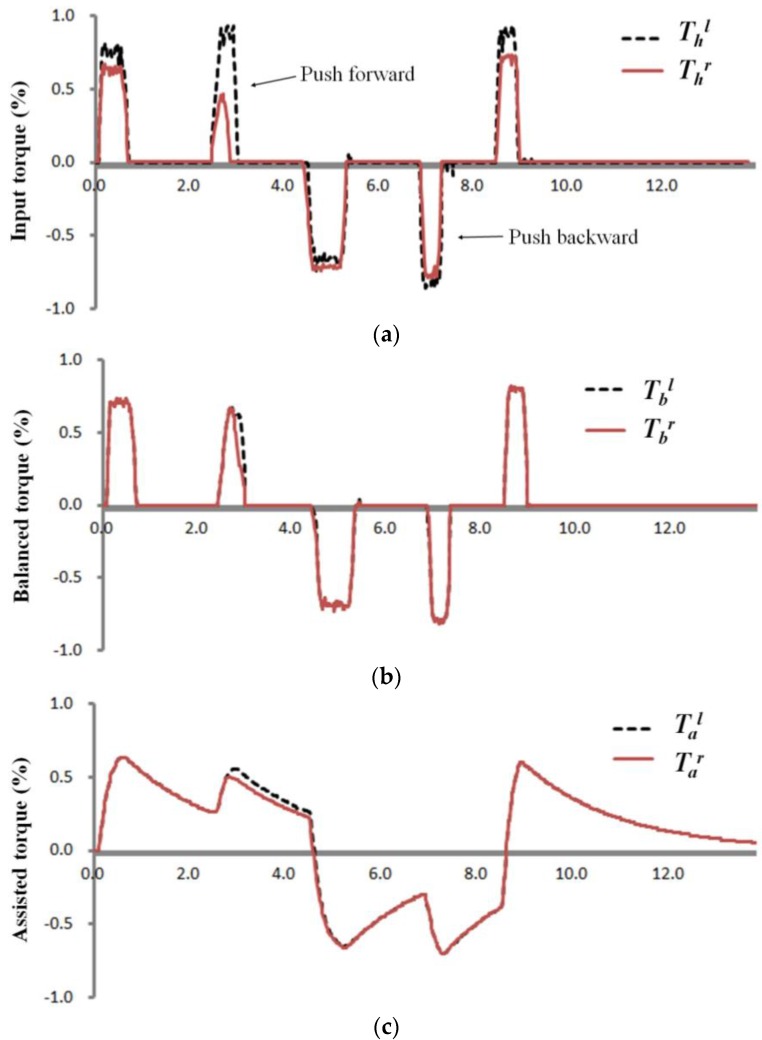
(**a**) Left and right input torque signals; (**b**) balanced torque signals; and (**c**) assist torque signals measured in HAPAW.

Figure 15 shows the sensor output measured by moving the handrim on the driving wheel backward and forward with the balanced torque and assist torque signals calculated from the sensor values. Each signal was acquired using a Bluetooth wireless interface between a PC and the driver as shown in Figure 9b. Torque applied on the handrim when propelling the wheelchair can become unstable because of the instability of the user’s posture, differences in left and right arm strengths, and mechanical imbalance of the wheelchair. As shown in Figure 15a, the input torque of the user applied to the handrim is unstable, and degradation of wheelchair driving performance can occur when this is delivered directly to the driving controller. Figure 15b shows the balanced torque waveform of the input torque corrected using the torque balance algorithm applied in this study, which considers input torque proportion and time differences. As a result of signal correction, instability between the left and right user-provided torques in Figure 15a was considerably stabilized, and it can be seen in Figure 15c that left and right output torques were also generated stably from the driving controller when balanced torques were applied. From the driving test results, it is clear that the developed non-contact torque sensor has enough applicability to be used in the development of driving wheels in HAPAW.

## 4. Discussion

In HAPAW, sensors that can detect handrim movement have been used in various methods to detect the driving intentions of users. A commercially available HAPAW assists drivability without receiving driving intention input from handrims [20]. This type of HAPAW assists in providing electric torque by detecting wheelchair movement. Additionally, as continuous driving is possible, the driving part is detachable, and high-speed driving is possible. This is a HAPAW preferred by wheelchair users who are highly active and have sufficient arm strength. Another type of HAPAW developed recently by Alber also enables high-speed driving and supports manual wheelchair users who have high levels of activity. HAPAW that enables continuous driving without stopping with one push of the handrim is also being researched [20,21], so it is evident that the main themes of current HAPAW development are high-speed driving and continuous driving.

±8° is too wide a range of motion (ROM) for handrims. The ROM of handrims in another commercial HAPAW is approximately ±2~3°, which is much smaller than in our prototype. The driving wheel prototype used in this study was manufactured for research, and a wide angle was applied to obtain sufficient data from flexible handrim movement. However, future research is planned that will use a very small range. The dead zone that occurs when the handrim spoke and the bar are separated is a region that includes a great amount of information related to the driving intentions of users, and it is necessary to minimize this clearance. Noise occurs when the dead zone is decreased owing to vibration or impact applied on the handrim, and it is expected that this can be sufficiently eliminated by software components such as low-pass filters.

The amplitude of the sensor signal decreases when the ROM of the handrim decreases. The sensor moving angle of the designed driving wheel is larger than the handrim moving angle because of the hinge structure of the bar, and the sensor moving angle can be adjusted depending on the hinge location. Therefore, the sensor signal size can be maximized by adjusting the hinge location even if the ROM is reduced. In this case, the linearity of the sensor signal deteriorates compared to Figure 10, but it is expected that it can be used in actual applications with simple software correction.

The springs for the handrims used in the prototype discussed here apply commercial products that have known spring characteristics. Modification of the spring constant and suitability of the spring for mass production are required for commercialization of this research. The prototype was developed to minimize mechanical friction and loss; assembly is not easy, and vibration occurs when releasing the handrims. Application of a structure with a spring of proper stiffness that can reduce handrim vibration is required for commercialization.

The handrim angle shows non-linearity in the 1°–2° and 7°–8° intervals as the magnet attached on the end of the bar rotates as shown in Figure 10. Owing to this influence, test torque values do not rise in the 7°–8° interval but slightly decline, which is different from the simulated torque values in Figure 13b. Therefore, it is expected that the uncertainty of the HAPAW can be reduced by decreasing the ROM of the handrim.

The battery can be considered as the core component related to driving distance and total weight of a HAPAW. Existing HAPAW mainly use lithium ion batteries or lithium iron phosphate batteries, which are light and have outstanding discharge performance. Lithium batteries have outstanding performance but high price, which is the main factor increasing wheelchair cost. The performance and price of the motor must also be considered significant as well as the battery. One of the most important factors of wheelchair cost determination is the level of health insurance supported by the government, and core components, such as motors and batteries, must be selected considering this fact.

## 5. Conclusions

PAWs detect the driving intentions of users and can assist mobility, which is very useful for the elderly and disabled, who lack strength. It is important in HAPAW to obtain the driving intention signal of a user from the rotating handrim and to deliver this easily and accurately to the fixed controller. A fixed Hall IC detecting the magnet on a rotating part was used in this study to design a wheel mechanism and sensor that detect handrim movement easily and simply and to manufacture a prototype. The torque measured from the designed driving wheel was simulated, and a prototype was manufactured for verification. The driving wheel prototype balances uneven input signals based on the user driving intention to generate stable assist torques, and results confirmed the feasibility of using it in HAPAW. Performance of the manufactured prototype must be improved because an intuitive interface for users is important in PAW. To this end, more elaborate torque sensors must be researched, and tests and research on durability and impact stability are planned in preparation for commercialization. In addition, additional research and investigation on the rotation of a wheelchair on flatlands and driving on slopes is required to be performed.

## Figures and Tables

**Table 1:** The specifications of the developed HAPAW.

**Wheel Diameter**		24 inch
**DC Motor**	**Rated Power**	80 W
	**Rated Speed**	7000 rpm
	**Weight**	1 kg
**Gear Ratio**		1/115
**Max. Wheel Speed**		61 rpm
**Battery**		24 V, 8 Ah
**Max. HAPAW Speed**		6 km/h

## Supplementary Materials

The supplementary materials are available online at http://www.mdpi.com/1424-8220/16/8/1251/s1, two videos for Figure 14 related to the experiments with different users are added as supplementary materials.
